# Leadership and work-life-balance beyond traditional gender roles

**DOI:** 10.1192/j.eurpsy.2026.10272

**Published:** 2026-07-31

**Authors:** A. Riecher-Rössler

**Affiliations:** Medical Faculty, University of Basel, Basel, Switzerland

## Abstract

**Introduction:**

More women than men enter psychiatry in the meantime, but the proportion of female leaders is still very low

**Objectives:**

In this interactive session you will get to know

- some data on gender and career in medicine and psychiatry,
- on career obstacles for women including their structural, but also psychological reasons, esp. the influence of traditional gender roles,
- on the negative consequences for both female and male psychiatrists and their patients, and
- on alternative solutions.

**Methods:**

overview and discussion

**Results:**

In order to promote careers and work-life balance for both genders in psychiatry we need

- structural reforms
- Mentoring with early start and ongoing coaching
- Questioning of traditional gender roles and promoting role models

**Conclusions:**

Especially in psychiatry we should aim at integration of living and working for both genders.

**Disclosure of Interest:**

None Declared

